# Detection and Classification of Malicious Flows in Software-Defined Networks Using Data Mining Techniques

**DOI:** 10.3390/s21092972

**Published:** 2021-04-23

**Authors:** Marek Amanowicz, Damian Jankowski

**Affiliations:** 1 NASK National Research Institute, 01-045 Warsaw, Poland; 2 Ministry of National Defense, 01-045 Warsaw, Poland; damian.jankowski@wat.edu.pl

**Keywords:** software-defined network, flow features, data mining, flow classification, Mininet, OpenDaylight

## Abstract

The increasing availability of mobile devices and applications, the progress in virtualisation technologies, and advances in the development of cloud-based distributed data centres have significantly stimulated the growing interest in the use of software-defined networks (SDNs) for both wired and wireless applications. Standards-based software abstraction between the network control plane and the underlying data forwarding plane, including both physical and virtual devices, provides an opportunity to significantly increase network security. In this paper, to secure SDNs against intruders’ actions, we propose a comprehensive system that exploits the advantages of SDNs’ native features and implements data mining to detect and classify malicious flows in the SDN data plane. The architecture of the system and its mechanisms are described, with an emphasis on flow rule generation and flow classification. The concept was verified in the SDN testbed environment that reflects typical SDN flows. The experiments confirmed that the system can be successfully implemented in SDNs to mitigate threats caused by different malicious activities of intruders. The results show that our combination of data mining techniques provides better detection and classification of malicious flows than other solutions.

## 1. Introduction

A traditional communication network comprises interconnected and individually configured devices for forwarding data packets. This has a few limitations related to the flexibility of packet forwarding and network management, as well as inhibiting the introduction of new, more effective mechanisms. The increasing availability of mobile devices and applications, the progress in virtualisation techniques, and advances in the development of cloud-based distributed data centres has significantly stimulated the growing interest in the use of software-defined networks. An SDN decouples the control plane from the data plane, improving the flexibility and automation of network functions; creates favourable conditions for introducing innovations; and leads to a reduction in the SDN’s operating costs.

Although the implementation of software-based technology in wired networks is relatively easy and frequent, it also has benefits in the wireless domain [1]. For example, it enables better collaboration between access points in order to reduce radio-specific problems and enhance wireless network security.

The SDN architecture can also be successfully used in other areas. For example, the recent work on many-core systems-on-a-chip (MCSoCs) considered adopting the SDN concept to design low-cost, high-performance architecture for aperiodic and low-duty-cycle traffic between cores [2]. An MCSoC in smartphones or IoT devices has a huge number of processing cores and many memories connected to one another by an on-chip network; therefore, the introduction of the SDN architecture may significantly improve network management performance.

**Figure 1:**
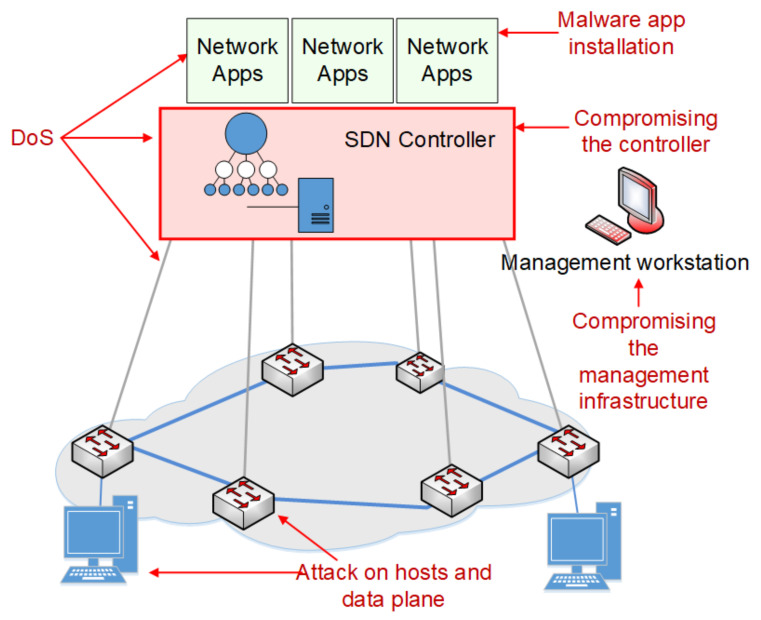
Possible attacks and threat vectors targeting SDN components.

However, SDN technology has many vulnerabilities that can be exploited by an attacker to breach network security, as discussed by Kumar and Gupta [3]. Figure 1 shows the possible attacks and threat vectors targeting different components of the SDN architecture.

Cyber-attacks can exploit, for example:

- An incorrect configuration;
- The operating system’s kernel errors of SDN controller;
- Incorrect permissions;
- The insufficient validation of input data;
- Software coding mistakes, e.g., a buffer overflow.

A basic threat is taking over control of the SDN driver, which may compromise the entire SDN. The attacker has unauthorised control over the network devices. The level of vulnerability to such attacks is mostly conditioned by the implementation of an SDN driver.

There are some solutions to detect and neutralise such threats, e.g., an intrusion detection system (IDS), an intrusion prevention system (IPS), or SDN controller replication mechanisms, as proposed by Gonzales et al. [4]. Lee et al. [5] discussed SDN security issues resulting from attacks on the northern interface, involving taking control of network applications or introducing malicious software. Such attacks cause illegal actions, e.g., the manipulation of flow rules, redirecting packets to an unauthorised recipient, or blocking selected traffic flows. An attack can also be targeted at the southbound interface by exploiting vulnerabilities of the protocols used, which enables the attacker to monitor or interfere in the exchange of messages between the controller and network devices. The SDN controller can be treated as a single point of failure, and therefore, it is a particularly attractive target for denial-of-service (DoS) attacks from both northbound and southbound interfaces. An attack from the northbound interface on one application can negatively affect another application that is not being directly attacked and, consequently, may, for example, introduce many conflicting flow rules for many applications. In the case of reactive flow entries, where each packet that does not match the existing entries in the flow table is forwarded to the controller, the generation of a huge number of malicious flows can overload the SDN controller and, consequently, disable network control.

Several practical solutions have been proposed to secure SDNs. For example, Scott-Hayward et al. [6] discussed the challenges in securing an SDN from a persistent attacker and proposed a holistic approach to the development of the SDN security architecture. They also identified research directions essential for providing network security. A state-of-the-art solution proposed to secure SDNs was discussed in [7]. The authors classified security solutions in terms of SDN layers/interfaces, security measures, simulation environments, and security objectives, as well as providing their own view on potential security requirements and key enablers for securing SDNs.

However, standards-based software abstraction between the network control plane and the underlying data forwarding plane, including both physical and virtual devices, provides an opportunity to significantly increase network security by using native SDN features, as discussed by Shin et al. [8] and Yoon et al. [9].

We propose to take advantage of such features, mainly related to the aggregation of various statistics from network devices, the openness for the implementation of new applications enabling the proper processing of traffic data, and their integration with network control mechanisms. We also recommend using the data mining technique (DMT) to detect and classify malicious flows in the SDN data plane. The use of DMTs is more common in detecting unauthorised activities in complex, multi-service information systems. DMTs also allow improving the efficiency and flexibility of intrusion detection, detecting new types of threats, highlighting symptoms of a specific attack, and precisely distinguishing between malicious and legal activities.

Therefore, this study’s aims were twofold:

1. To present a comprehensive solution that can be successfully used in the SDN environment to mitigate threats caused by different malicious activities of intruders;
2. To demonstrate that our combination of data mining techniques provides better detection and classification of malicious flows compared with other solutions.

This study continues our preliminary works on assessing the rationality of using transformation techniques such as ICA and PCA to reduce the features space and processing time [10] and exploring methods of generating both normal and malicious flows that can be used to evaluate various SDN-based intrusion detection systems [11].

Briefly, the main contributions of this paper are as follows:

- The elaboration of a flow rule generation mechanism that allows for the online adjustment of the granularity of rules to the current traffic volume and obtains a good balance between the number of captured features to precisely identify network traffic and the controller protection against flooding;
- The extension of flow classifier functions to enable the examination of different classification methods by the appropriate selection of parameters for the technique used and their values, as well as the attributes of the learning phase;
- The presentation of the Monitoring and Detection of Malicious Activities in SDN (MADMAS) system deployment in a virtual environment that allows for its examination in conditions similar to the real ones;
- The demonstration of the performance of MADMAS system alternatives and their effectiveness in malicious flow detection and classification compared with other solutions described in the literature.

The rest of the paper is structured as follows. Section 2 presents a brief overview of an available solution that enables the detection of malicious activities in SDNs, with particular emphasis on the data mining techniques used. Section 3 provides an overview of the system architecture, also showing the techniques used at individual stages of flow detection and classification and the mechanisms of flow rule generation and flow classification. Section 4 describes experiments that confirmed the MADMAS system’s effectiveness in the detection and classification of malicious flows under conditions that reflect typical SDN activities. The paper concludes with some remarks and proposals for the future.

## 2. Related Work

Different types of intrusion detection systems (IDSs), commonly used to monitor networks or systems for malicious activities, have been discussed previously [12]. Such systems can be placed at strategic points within a network to monitor traffic flows on the network (so-called network IDS (NIDS)) or at individual hosts or devices to monitor inbound and outbound packets from a device (so-called host IDS (HIDS)). The most well-known IDSs are signature-based systems that recognise incorrect traffic patterns and anomaly-based systems, detecting deviations from a model of expected normal traffic, which often apply machine learning techniques. 

Signature-based intrusion detection systems define a set of attack patterns (signatures) and establish a threshold of similarity to the predefined pattern, enabling the triggering of an alarm. Signature-based systems are often used because they are efficient at sniffing known attacks; however, they may be ineffective in cases of completely new attacks for which no pattern has been defined. 

Anomaly-based intrusion detection systems measure the present state of network traffic in order to detect patterns that deviate from normal activity. Malicious activity detection is based on features extracted from traffic flows, both at the data layer, as shown by Umer et al. [13], and at the application layer, as shown by Kozik et al. [14]. Such systems are appropriate for detecting new attacks or attacks that have been deliberately assembled to avoid detection; however, they may generate larger numbers of false positives compared with signature-based systems. Bhuyan et al. reviewed a large number of network anomaly detection methods and systems, in terms of the computation techniques used [15], and Boriah et al. discussed proximity measures that can be used for pattern recognition [16].

Anomalies in traffic flows can be detected in several ways, most often with machine learning and data mining. Machine learning is more common in systems for supporting computer emergency response teams during threat detection [17,18]. Data mining, as presented by Buczak and Guven [19] or Dua and Du [20], creates new possibilities for detecting new types of threats and contributes to improving the efficiency and flexibility of anomaly-based IDSs. The application of a DMT for flow processing highlights specific symptoms of an attack, while its use for classification clearly distinguishes an attack from legal activity [21].

The deep packet inspection (DPI) technique, implemented in specialised network components, is often used to extract diagnostic features from traffic flows [22]. This technique enables the collection of detailed data on traffic flows; however, processing all packets usually requires significant computing resources. Another approach is to sample only selected flow parameters (so-called flow-based IDS), e.g., the number of bytes transferred or a flow duration or L3 and L4 protocols. The extracted parameters do not allow for describing network activity exactly like DPI, but the computing resources might be significantly reduced.

Predefined sets of test data, such as KDD99 Cup DARPA [23] or NSL-KDD [24], are often used to assess the efficiency of feature detection and processing algorithms. These sets contain data vectors representing network activities with their diagnostic features that are labelled as either normal or malicious with exactly the specific attack type, i.e., denial of service (DoS), scanning, unauthorised access to a local machine (R2U), or illegally obtaining the root’s privileges (R2L). 

A number of studies have elaborated security solutions enabling the effective detection of anomalies in a software-defined environment. Some examples of the proposed methods and systems are discussed below.

Mehdi et al. [25] propose a mechanism based on evaluation of the first packet sent to the controller for the identification of abnormal behaviours at the underlaying network layer. They used the threshold random walk with credit-based rate-limiting (TRW-CB), maximum entropy detector, rate-limiting, and network anomaly detection (NETAD) algorithms, all implemented in the SDN controller. The type of read parameters and matched fields of the flow depend on the type of anomaly detection algorithm used. This approach assumes that it is possible to detect selected attacks without the need to analyse all packets of the flow. The method focuses on detecting port scanning and L3/4 DoS attacks. The disadvantage of the method is the inability to detect application layer attacks. Normal traffic was collected during the operation of an actual SDN, while unauthorised traffic was generated in the laboratory environment. Both classes of traffic are combined into one dataset; however, the authors do not depict the procedure of test dataset generation.

A mechanism for information security management in SDNs, as proposed by Dotcenko et al. [26], combines algorithms of statistics collection, data processing, and decision making implemented in the SDN controller, and it uses a fuzzy logic technique for inference. The TRW-CB and rate-limiting algorithms are used to collect diagnostic features of the network traffic. If suspicious traffic is identified, the threat level is expressed on a three-level scale: low, medium, or high. The limited scope of attack detection and the lack of advanced methods for verification of the effectiveness of the proposed solution are the basic limitations. In addition, the description of the statistics collection module is generic. The authors do not depict a measurement procedure of the network traffic parameters, but only specify the potential functions of the module.

An example of the use of data aggregation and traffic profile collection in the SDN controller is presented in [27]. The authors use OpenFlow and sFlow protocols to collect network traffic parameters. Aggregation of network traffic parameters involves periodically sending requests from the SDN driver to network devices and receiving network statistics, which is closely related to packet forwarding. 

Another interesting approach [28] involves the use of the sFlow protocol to sample network traffic parameters, which allows the separation of sampling from packet forwarding. This, in turn, enables extracting network traffic parameters without burdening the SDN driver’s resources, but it requires the implementation of an additional protocol. The proposed method allows the detection of DDoS L3–L4 attacks, worm propagation, and port scanning. To detect unauthorised activities, the system measures the entropy changes of four network traffic characteristics for a given network device: source and target IP addresses, and source and target TCP/UDP ports. Based on the changes in the entropy level of these characteristics, it is possible to identify symptoms corresponding to specific classes of the unauthorised activities. A significant decrease in the entropy of destination IP addresses and destination TCP/UDP ports is considered a symptom of a DDoS attack. The presence of worm propagation is evidenced by a significant decrease in the entropy of source IP addresses and destination TCP/UDP ports. Scanning specific host ports reduces the entropy of destination and source IP addresses and increases the entropy of source TCP/UDP ports. The system was tested with real traffic and optimised to handle high bit rates, which, however, limited the level of detail of the analysis of unauthorised activities. Thus, the system enables the detection of unauthorised activities with regard to the entire flow table but without indicating the specific attack class associated with a given flow.

Braga et al. [29] proposed a self-organising map (SOM) as a mechanism for detecting unauthorised activities. The system consists of three main components: a flow collector, a feature extractor, and a classifier. These three components periodically sample the parameters of entries in flow tables, convert flow parameters into diagnostic features, and detect attacks, respectively. SOM-based analysis, which is performed using data on five traffic flow parameters (i.e., average number of packets, average duration of flow, percentage of pairs of flows, increase in the number of individual flows, and increase in the number of flows with different ports), enables a high rate of detecting DDoS L3/4 attacks from botnets and a low rate of false alarms. Unfortunately, diagnostic features are determined for the entire flow table of the network device, so it is not possible to detect single malicious flows; however, it is possible to indicate a network device through which such traffic is forwarded.

The system presented in [30] enables detecting DoS, probe, R2L, and U2R attacks in an OpenFlow-based SDN with the use of the J48 decision tree (the implementation of a C4.5 tree) based on the information gain measurement [31]. The traffic parameters are determined in accordance with the diagnostic features of the NSL-KDD set. The binary bat algorithm (BBA) [32], which performs feature selection, is an example of a swarm intelligence (SI) algorithm, which refers to the behaviour of bats [33]. The authors do not specify a mechanism for obtaining diagnostic features for classification; therefore, the system was tested using the ready-made NSL-KDD dataset.

Le et al. [34] present a system for detecting malicious activities in an OpenFlow-based SDN using port mirroring, where packets are additionally copied to the interface of a switch connected to the intrusion detection system (IDS). In packet inspection, 25 diagnostic features are determined and divided into two groups: basic features, represented by network connection parameters, and derived features, called network traffic characteristics, determined within a specific time window, reflecting the degree of similarity of different TCP connections. Vectors of features are used for the C4.5 classification algorithm, which is a variant of the decision tree [35]. Classification results, providing information about the detection of unauthorised activities, are sent to the SDN driver, which introduces a flow rule that blocks the traffic identified as illegal. The system was tested using the KDD99CUP dataset, as well as in the real environment. Three types of DoS attacks and eight types of probe attacks were used in experiments conducted in the real network; however, their generation was not described. This approach can be considered a hybrid one in which the SDN’s capabilities are used only for forwarding packets to the appropriate IDS and then introducing new flow rules in response to identified threats. Although the system architecture would allow the collection of data on the application layer and packet payload, such a mechanism was not implemented.

Tang et al. [36] propose a mechanism applying deep learning for detecting malicious flows in an OpenFlow-based SDN. Selected parameters of network flows are collected from OpenFlow switches and then transferred to the component located in the SDN controller responsible for flow-based anomaly detection. The deep neural network (DNN) model was learned with the NSL-KDD dataset. The mechanism was tested in terms of the detection of DoS, RL2, U2R, and probe attacks, as well as its comparison with other classification techniques such as J48, naive Bayes (NB), tree and random forest, NBTree, multi-layer perceptron (MLP), and support vector machine (SVM). The obtained results indicate a relatively low efficiency of the DNN-based mechanism, as well as other machine learning methods, which may indicate a too-narrow scope of the experiments and non-optimal selection of the parameters of the classification.

The above solutions focus on a comparison and evaluation of the machine learning techniques for anomaly detection. By contrast, Querioz et al. [37] proposed the practical implementation of traffic measurement in OpenDaylight-based software-defined networks. The authors focused on online fine-grained measurements of throughput at flow, port, link, path, and switch levels. They applied big data streaming tools to monitor the SDN bandwidth use, which supports traffic engineering activities and also enables the detection of DoS flows. 

The approach proposed by Tuan et al. [38] focused on using machine learning to mitigate DDoS attacks, especially TCP-SYN and ICMP flood attacks, in SDN-based internet service provider (ISP) networks. A lightweight and fast machine learning algorithm based on a k-NN that facilitated real-time operations was used to detect and mitigate attack traffic by tracing back the IP sources of attack, achieving a trade-off between accuracy and system capacity. The authors also proposed a method of optimising the monitoring window time for improvement in the mitigation algorithm efficiency.

An interesting approach to solving challenging problems in using legacy datasets, such as the KDD’99, for anomaly detection in the SDN environment is shown in [39]. The authors proposed a method of generating an attack-specific SDN dataset that can be publicly available. The new InSDN dataset included various attack categories that can occur in different elements of the SDN platform, such as DoS, DDoS, Botnet, web attacks, brute force attacks, malware, probes, or exploitation. In addition, some of them concern the SDN control plane. The authors also demonstrated the use of the InSDN dataset for evaluating some popular machine learning techniques for the detection of malicious activities.

Gomez-Rodriges et al. [40] present a wide overview of the literature on software-defined network-on-chip (SDNoC) use in MCSoC applications. They point out some security-oriented approaches supporting the SDNoC architecture that apply a security protocol for network configuration, as proposed by Ruaro et al. [41], or define security zones and apply an admission mechanism to accept new applications in a security zone, as presented by Ruaro et al. [42]. Security vulnerabilities, especially in the IoT context, arise due to exposure of the MCSoC infrastructure to DoS attacks by malicious users. An example of a low-cost mechanism for detecting the location of the attacker in the MCSoC and direction of the collision is presented by Chaves et al. [43].

In summary, a comparison of the basic features of the above-mentioned concepts of malicious flow detection in an SDN is presented in Table 1.

The functional architecture of the solutions presented above is similar, although they differ in the implementation of specific functions. In all solutions, traffic flow parameters obtained from network devices are collected in a dedicated component located in the SDN controller. Further processing of the collected features and detection of malicious actions take place in the components communicating with the SDN controller or placed directly in it. They enable detecting selected types of unauthorised activities, such as DoS, DDoS, port scanning, or attempts to propagate malware. Only those presented in [30] and [36] enable the detection of U2R and U2L attacks; however, they do not apply to mechanisms enabling the acquisition of information from the application layer. The solution presented in [34] uses the deep packet inspection (DPI) technique, implemented by forwarding the packet stream to the IDS, which is a form of the middle box between the data plane and the SDN driver, but the range of detected attacks includes only DoS and probing. None of the solutions presented allows the detection of DoS L7 attacks. In the case of such attacks, it is desirable to obtain flow parameters from the application layer and to distinguish them at the transport layer connections, although OpenFlow implementations have a limited ability to inspect package contents. Only the system described in [30] applies the feature selection mechanism, and the feature transformation technique is not used in any solution.

## 3. MADMAS Architecture

### 3.1. Architecture Overview

In a MADMAS system, the SDN controller acts as an intermediary platform for the centralised retrieval of traffic flow parameters from the switches. We assumed that measurements of traffic flow parameters, their processing, and flow feature selection should be performed in such a way and at such a level of detail that we can identify malicious hosts. Furthermore, the traffic measurement and feature processing mechanisms should use the native functions and protocols of the SDN, and the use of other mechanisms and protocols that are not part of the SDN environment should be limited.

**Figure 2:**
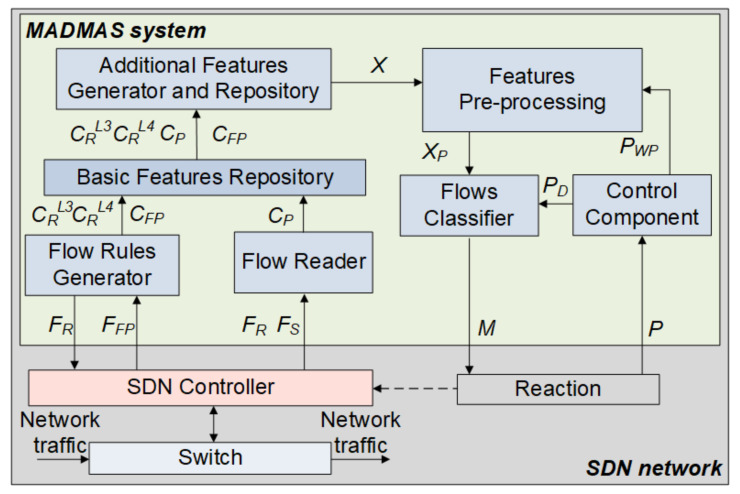
MADMAS architecture. The symbols used: *F_R_*—flow rule; *F_FP_*—first packet in a flow; *C_FP_*—features of *F_FP_*; *F_S_*—flow statistics; *C_P_*—basic flow features; *X*, *X_P_*—input vectors before and after pre-processing, respectively; *C_R_^L^*^3^, *C_R_^L^*^4^—attributes of flow granularity.

The MADMAS system architecture, presented in Figure 2, consists of seven main components: a flow rules generator (FRG), flow reader (FR), basic features repository (BFR), additional features generator and flows repository (AFG), features pre-processing (FPP), flow classifier (FC), and control component (CC). The system operates in the network environment, cooperating directly with the SDN controller.

The FRG generates flow rules to ensure packet transfer over the network between source and destination nodes. The flow rules granularity technique allows us to distinguish sessions and connections. Incorporating a mechanism that allows for dynamic adjustment of the granularity of the flow rules to the current traffic volume enables a good balance between the number of captured features to correctly identify the network traffic and the controller protection against flooding. The FRG is also responsible for collecting application layer data from the first packets of flow and provides information about the reduction in flow granulation. A detailed description of this component is given in Section 3.2.

The FR performs tasks related to the sequential reading of the contents of flow tables and extraction of data from flow rules (flow input port, source and destination addresses, layer 4 protocols, and source and destination TCP/UDP ports) and from flow statistics (maximum flow duration, number of bytes sent/transferred in the source/destination direction with or without the TCP PSH flag, number of packets sent in the source/destination direction with a TPC PSH flag set). For each composition of such data, the set of basic flow features CP is defined and stored in the BFR for further analysis. The FR is also responsible for the generation of application layer features. The UDP flows are taken directly from the payload of the first packet. For TPC flows, the application layer data are passed after the three-way handshake process. Therefore, the FR uses the PSH flag to distinguish such flows.

The additional features generator and repository is responsible for additional feature specification based on the basic features and content of flow tables. A set of additional features contains complementary data that reflect the interrelation of flows, changes in the value of some of their attributes, as well as data enabling the differentiation of traffic classes. This helps to increase the effectiveness of malicious activity detection, such as the maximum value of the flow coefficient with different or the same ports, the maximum value of the flow factor for a given target host, the maximum value of the single flow coefficient, the maximum value of the flow repetition coefficient, and maximum values of the layer 3 and layer 4 flow reduction coefficient. Both additional and application layer features are stored in the repository for further use in flow classification. The features pre-processing component carries out the initial phase of data mining. Based on the set of vectors X, which represent application layer data gained from the AFG, this component creates a set of vectors X_P_ containing selected features enabling effective flow classification. It comprises four 4 modules responsible for:

- The processing of application layer data with a text mining technique that includes input data tokenisation, n-gram analysis of tokens, features pruning, and features transformation using independent component analysis (ICA);
- The normalisation of the features for the unification of the numerical ranges of their values;
- The linear transformation of features with principal component analysis (PCA) in order to highlight specific aspects of the data;
- Feature selection for flow classification.

The transformation of the string of ASCII characters representing the application layer data into a set of tokens creates input data for n-gram analysis, which allows for the creation of a feature space for a string by counting occurrences of substrings consisting of n tokens. The result of n-gram analysis is a vector that defines the frequency distribution of the substrings for each string representing application layer data. The token occurrence frequency is determined by the TF-IDF method [44]. The result is a vector containing the weight of words occurring within the application layer data. The set of vectors can contain a large number of features, and therefore, additional processes are implemented to reduce their elements. The first reduction process removes tokens for which the TF-IDF value is outside the given frequency range of occurrence. Thus, the limited set of vectors is again reduced with ICA transformation [45].

The normalisation of features aims at achieving a coherent dataset and leads to unification of the numerical ranges and values of the data. The normalised vectors of the features are further subject to PCA transformation for feature space reduction. During the selection, a set of the most significant features contained in the vectors of reduced dimensionality is created, which is then used for flow classification.

The flow classifier carries out tasks related to malicious flow detection and assigns each malicious flow an appropriate label, representing a class of specific illegal activity. The outcomes of classification can trigger reaction procedures, including the introduction of new flow rules to eliminate identified threats. In the present version, the FC can be configured directly for flow classification with a predefined technique or for the examination of different classification methods by appropriate selection of the parameters of the technique used and their values, as well as training attributes. A detailed description of the FC is given in Section 3.3.

The control component enables the system’s operator to introduce modifications/changes to a technique used for flow classification in order to obtain an accepted level of system effectiveness. 

### 3.2. Flow Rules Generator

**Figure 3:**
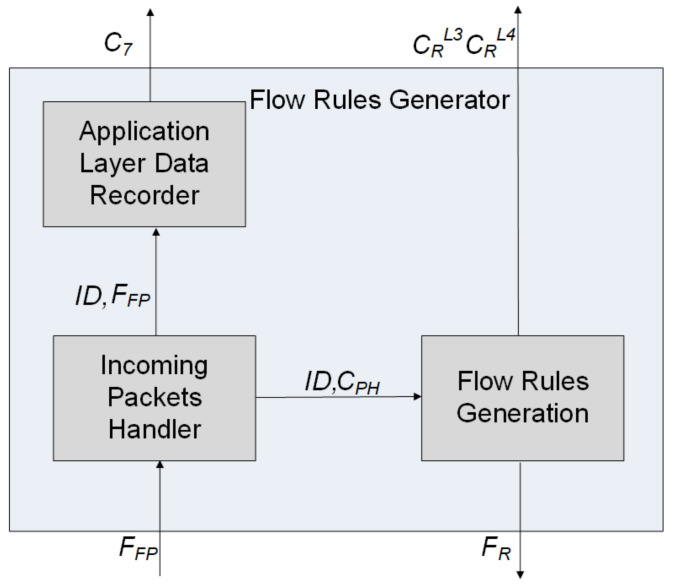
Structure of a flow rules generator. The symbols used: *F_R_*—flow rule; *F_FP_*—first packet in a flow; *C*_7_—application layer data of *F_FP_; ID*—identifier for joining *F_R_* and *C_FP_*; *C_PH_*—features from packet header; and *C_R_^L^*^3^, *C_R_^L^*^4^—attributes of flow granularity reduction.

The flow rules generator, whose internal structure is shown in Figure 3, consists of three modules: incoming packets handler (IPH), application layer data recorder (ALDR), and a flow rules generator (FRG). 

The IPH module receives the first packets *F_FP_* sent by the controller from flows for which no match rules *F_R_* are found, and it retrieves the information necessary to create flow rules as well as to obtain application layer data. A copy of the unprocessed application layer data *C_L_*_7_, together with the generated identifier *ID* used to associate data with the flow, is sent to the ALDR module for further feature selection. The application layer data of the UDP flow are already contained in the payload of the first packet transferred to the IPH module. However, for the TCP flow, application layer data are transferred only after establishing a connection (three-way handshake) between source and destination nodes, so the first TCP packet cannot be used for such identification. To resolve this issue, additional differentiation of flows is introduced by using the TCP push flag that enables forwarding the TCP packet to the controller, together with application layer data. 

The FRG module creates flow rules *F_R_* based on *C_PH_* data obtained from the packet header and the flow *ID*. A flow rule contains the identifier of the flow (*ID*), rules of the flow processing (*A_n_*) determining the PFD output port at which packets are forwarded, and the set of flow matching attributes:

(1) $$F_{R}=\left\{ID,\;P_{in},\;IP_{src},\;IP_{dst},p_{src},\;p_{dst},\;P_{L4},\;psh,\;A_{n}\right\}$$

where *P_in_* is the PFD input port, *IP_src_* is the source IP address, *IP_dst_* is the destination IP address, *p_src_* is the L4 input port, *p_dst_* is the L4 output port, *P_L_*_4_ is the L4 protocol, and *psh* is the status of the PSH flag.

The flow rule is removed if no new packet corresponding to it is received within the time frame *t_idle_* > 0. There are a number of benefits of reactive flow rule removal, including the ability to determine the flow duration, a reduction in the size of flow tables, and an increase in the level of flow granularity.

Flow rules have a specific level of granularity that allows us to identify the type of network traffic and distinguish sessions and connections. Increasing flow granularity allows more detail in capturing traffic features. In contrast, a reduction in flow granularity allows us to reduce the number of packets sent to the controller; however, this leads to a decrease in the level of details in measuring traffic characteristics. Increasing the granularity of flows results in passing more packets to the controller, which must be processed to implement flow rules. This can result in increased consumption of controller resources, which, in turn, can increase the delay in packet processing. A situation in which packets are sent to the controller in a number significantly exceeding the normal level of network traffic is interpreted as controller flooding. To avoid such adverse events, the regulation mechanism of flow granularity was introduced, as shown in Algorithm 1, which generates the values of granularity reduction attributes that are submitted to the basic feature repository.
**Algorithm 1** Reduction in flow granularity**Input arguments:** σ_L4_: L4 reduction threshold;  σ_L3_: L3 reduction thresholdT_IP_: set of IP addresses of packets sent to the controller T_P_; set of ports in the packet forwarding device (PFD) **Loop**
**for each**
*IP_i_* in *T_IP_*
**determine**$\Delta P_{i}^{L4}$$\;\mathrm{for}\;IP_{i}$
**if**
$\Delta P_{i}^{L4}$ from $IP_{src}>\sigma_{L4}$
**read**
$R_{C}^{L4}$ for $IP_{i}$
**determine**
$t_{hard}^{L4}$ based on $\Delta P_{i}^{L4}$, $R_{C}^{L4}$
**determine**
*ID,*
$A_{n}$
**determine**
$F_{R}^{L4}$ based on *ID*, $t_{hard}^{L4}$, $A_{n}$
**introduce**
$F_{R}^{L4}$ to PFD **determine**
$P_{C}^{L4}$ for $IP_{i}$
**determine**
$C_{R}^{L4}$ based on $\Delta P_{i}^{L4}$, $R_{C}^{L4}$, $P_{C}^{L4}$
**introduce**
$\left\{ID,C_{R}^{L4}\right\}$ to BFR **update**
$P_{C}^{L4}$ for $IP_{i}$
**end**
**for each**
*P_i_* in *T_P_*
**determine**
$\Delta P_{i}^{L3}$ for $P_{i}$
**if**
$\Delta P_{i}^{L3}$ from $P_{in}>\sigma_{L3}$
**read**
$R_{C}^{L3}$ for $P_{i}$
**determine**
$t_{hard}^{L3}$ based on $\Delta P_{i}^{L3}$, $R_{C}^{L3}$
**determine**
*ID,*
$A_{n}$
**determine**
$F_{R}^{L3}$ based on *ID*, $t_{hard}^{L3}$, $A_{n}$
**introduce**
$F_{R}^{L3}$ to PFD **determine**
$P_{C}^{L3}$ for $P_{i}$
**determine**
$C_{R}^{L3}$ based on $\Delta P_{i}^{L3}$, $R_{C}^{L3}$, $P_{C}^{L3}$
**introduce**
$\left\{ID,C_{R}^{L3}\right\}$ to BFR **update**
$P_{C}^{L3}$ for $P_{i}$
**end**
**endloop**
**Output arguments:**
$F_{R}^{L4}$, $F_{R}^{L3}$, $t_{hard}^{L4}$, $t_{hard}^{L3}$, $C_{R}^{L4}$, $C_{R}^{L3}$


The attributes are determined by the values of the packet parameters of the incoming flows in relation to:

- IP source address (*IP_sr_*): reduction at the L4 level;
- PFD input port (*P_in_*): reduction at the L3 level.

The flow rules take the following forms:

$F_{R}^{L4}=\left\{ID,\;P_{in},\;p_{src},\;p_{dst},\;A_{n}\right\}$: in the case of granularity reduction at the L4 level;

$F_{R}^{L3}=\left\{ID,\;P_{in},\;p_{dst},A_{n}\right\}$: in the case of granularity reduction at the L3 level.

The attributes of the flow granularity reduction at the L4 level are determined according to the following formula:

(2) $$C_{R}^{L4}=\frac{1}{1+e^{-\alpha \left(\Delta P^{L4\;}+P_{C}^{L4}+R_{C}^{L4}\right)}}$$

where α is the shape factor, Δ*P^L^*^4^ is the number of packets sent from the given *IP_sr_* address within t = 1 s, *P_C_^L^*^4^ is the total number of packets sent since the start until the end of reduction, and *R_C_^L^*^4^ is the number of previous reductions for the given *IP_sr_* address, *C_R_^L^*^4^ *∈* *(0,1)*.

The attributes of the flow granularity reduction at the L3 level are determined according to the following formula:

(3) $$C_{R}^{L3}=\frac{1}{1+e^{-\alpha \left(\Delta P^{L3\;}+P_{C}^{L3}+R_{C}^{L3}\right)}}$$

where *α* is the shape factor, Δ*P^L^*^3^ is the number of packets sent from the given port *P_in_* within t = 1 s, *P_C_^L^*^3^ is the total number of packets sent since the start until the end of the reduction, and *R_C_^L^*^3^ is the number of previous reductions for the given port *P_in_*, *C_R_^L^*^3^ *∈* *(0,1)*.

For a given flow rule, the additional parameter *t_hard_* is also determined, according to Equations (4) and (5), which defines the time for which the reduced flow rule is introduced:

(4) $$t_{hard}^{L4}=\frac{t^{L4}}{1+e^{-\alpha \left(\Delta P^{L4\;}+R_{C}^{L4}\right)}}$$

(5) $$t_{hard}^{L3}=\frac{t^{L3}}{1+e^{-\alpha \left(\Delta P^{L3\;}+R_{C}^{L3}\right)}}$$

where *t^L^*^3^ and *t^L^*^4^ are the maximum values of *t_hard_^L^*^3^ and *t_hard_^L^*^4^, respectively.

After time *t_hard_*, the reduced granularity flow is removed from the table, regardless of whether packets are being forwarded within this flow. This enables us to continue introducing flows of high granularity according to Equation (1).

### 3.3. Flow Classifier

**Figure 4:**
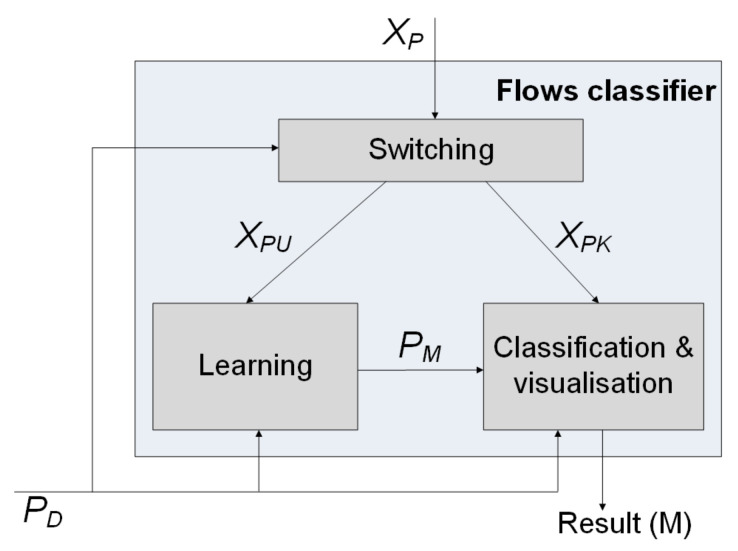
Structure of the flow classifier. The symbols used: *X_P_*—input vectors after features processing; *X_PU_*—input vectors for learning; *X_PK_*—input vectors for classification; *P_M_*—learned model parameters for classification and visualisation; and *P_D_*—hyperparameters for classification and visualisation.

The flow classifier (FC) performs tasks related to the detection of malicious flows in the SDN data plane using selected classification techniques. It is composed of three modules: switching, learning, and classification and visualisation (Figure 4). The switching module divides the set of features after pre-processing *X_P_* into the subsets *X_PU_* and *X_PK_*. *X_PU_* contains the vectors of input data for the learning phase of the selected algorithm, which is performed in the first stage of malicious flow detection. *X_PK_* contains the vectors of input data used for flow classification. The division of *X_P_* into *X_PU_* and *X_PK_* is determined by values of the *P_D_* parameters.

The learning module carries out the process of choosing the parameter values of the selected classification method using the *X_PU_* learning subset. Based on this, the *P_M_* model is built, which is used to predict a class of flows for a new pattern whose input arguments are not included in the learning dataset.

Proper malicious flow detection using the *X_PU_* subset of input data is performed by the classification and visualisation module, which executes two processes:

- The detection of unauthorised activity and assigning to it an appropriate label of malicious action;
- The visualisation of classification results.

The presented solution assumes that the following classification techniques can be used to detect undesirable flows:

- Multilayer perceptron (MLP) and radial basis function (RBF);
- Multipass self-organising map (MSOM);
- Learning vector quantisation (LVQ) and hierarchical LVQ (HLVQ);
- Support vector machine (SVM);
- k-Nearest neighbour (k-NN).

To ensure the effectiveness of unauthorised flow detection, the MADMAS system allows us to modify the values of *P_D_* parameters of the applied classification technique, the list of which is given in Table 2. The type of parameter and its value depend on the technique used, as well as on the value of the following attributes:

- *p_split_∈(0,1)*: learning/detection split ratio;
- *n*: number of learning cycles if cross-validation is applied;
- *P_M_*: parameters of a detection model.

The *P_D_* parameter values are defined by the MADMAS user, depending on the actual needs, and entered via the control module.

## 4. MADMAS Examination

### 4.1. Experimental Setup

**Figure 5:**
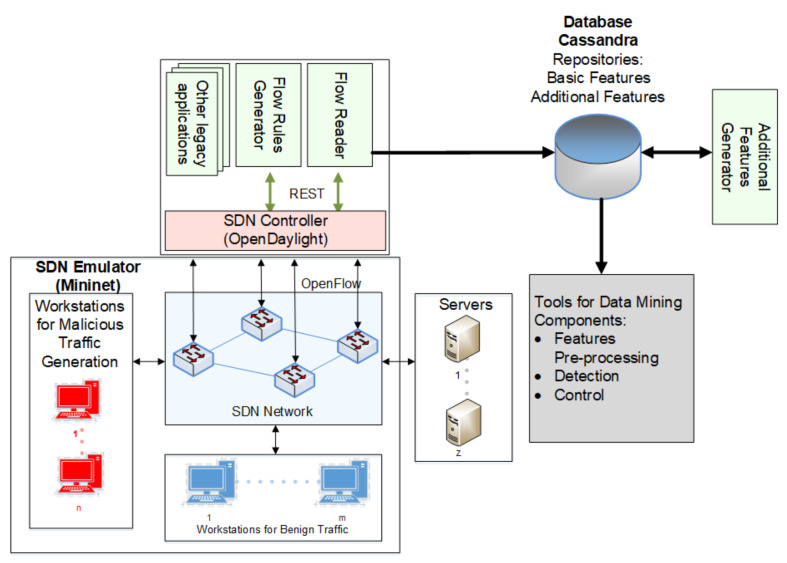
MADMAS testbed environment.

The aim of the study was to examine the effectiveness of MADMAS in the detection and classification of malicious flows under typical SDN traffic conditions. For this purpose, an experimental tested environment was developed containing an SDN emulator, an OpenDaylight (ODL) controller containing MADMAS components, and data centre servers, all implemented on a single server hosting some virtual machines, as shown in Figure 5. 

The flow rules generator was implemented as the OSGi network application in the ODL based on the OpenDaylight L2 switch project modification. The FR read the flow rules using ODL API REST messages. The NoSQL Cassandra database (column family database) was used to store datasets of basic and additional flow features. The specialised tools MATLAB, WEKA, and RapidMiner were used for the implementation of classification techniques. The control component contained a set of dedicated tools and scripts for the automatic change of parameters of individual methods. The Mininet platform was used as the SDN emulator. The data centre side was emulated by Metasploi Table 2 virtual machines. Traffic generators of normal as well as malicious traffic were implemented on separate virtual machines. 

To reflect typical SDN traffic conditions, five classes of flows were generated that represent both normal and malicious network activities:

- Normal (*N*): correct flows between clients and servers;
- Denial of service (DoS): actions aimed at making network resources unavailable to users;
- Probe (*P*): actions aimed at ports, vulnerabilities or version scans;
- Access by exploit (*A_E_*): actions enabling remote access to machines by exploiting vulnerabilities;
- Access by password guess (*A_PG_*): actions enabling access to remote machines through attempts of unauthorised login.

The list of applications used for traffic generation is presented in Table 3.

It was assumed that the generated traffic would be complex, preventing the direct detection of malicious flows. However, due to the complexity of real traffic, it was necessary to adopt some assumptions and simplifications that do not affect the credibility of the outcomes of system examination:

- Services indicated in Table 3 are running on the servers;
- Data are exchanged between servers and hosts;
- Hosts initiate normal and malicious traffic;
- Hosts do not cooperate with one another;
- Unauthorised and normal traffic is generated simultaneously on separate virtual machines, with the parameters presented in Table 4.

Normal traffic is generated using client applications according to the Poisson distribution, while malicious traffic is generated according to the normal distribution. Each class of unauthorised action has subclasses, which define the detailed course of action and type of tools or exploits applied for attacks targeted at a server or network resources. For example, Nping is used to generate a flooding attack that affects both the performance of the SDN controller and the available data plane resources and can cause delays in flow matching.

### 4.2. Testing Conditions

It was assumed that malicious flow detection was performed in off-line passive mode, i.e., the core detection process occurs after the completion of flow feature measurements on a data mining platform. Test data for individual methods were stored in the repository and read for experimentation. This approach allowed for a comprehensive study and comparison of the effectiveness of selected classification techniques, as well as indicating the most effective one, tailored to the specificity of traffic flows in the SDN. 

The detection and classification of malicious flows by MADMAS requires the introduction of a set of input vectors *X* to the FPP and FC. The MADMAS system was examined using repetitions of the learning processes, the so-called k-cross-validation, with different learning datasets. The input dataset was divided into *k* = 10 parts, of which *k* − 1 were used for learning. The procedure was repeated *k* times, changing the testing subset each time.

The following metrics were used to evaluate flow classification performance:

- Recall rate:

(6) $$TPR=\frac{TP}{TP+FN}$$

where *TP* is a true positive and *FN* is a false negative;

- Precision rate:

(7) $$PPV=\frac{TP}{TP+FP}$$

where *FP* is a false positive;

- F-measure (*F*_1_ score):

(8) $$F_{1}=2\frac{PPV\Delta TPR}{PPV+TPR}$$

In addition, the following time measures were used for system evaluation:

- Average execution time:

(9) $$AET=\frac{t_{x}}{n_{f}}$$

where *t_x_* is the cross-validation time and *n_f_* is the number of datasets used for cross-validation;

- Flow transfer delay (round-trip time):

(10) $$F_{RTT}=t_{r}-t_{s}$$

where *t_s_* is the time of sending the first packet and *t_r_* is the time of receiving the response.

The experiments presented below aimed at:

- Assessing the mechanism of granularity reduction;
- Identifying the most suitable technique for detection and classification of malicious flows in the SDN environment.

### 4.3. Flow Granularity Reduction 

The study of the flow granularity reduction mechanism was performed in two modes of the MADMAS system, i.e., with the mechanism on and off. In both cases, a source host generated ICMP packets with the given intensity *I_F_* for a set of receiving hosts. It was assumed that no flow rule existed for any generated packet, which forced it to be transferred to the SDN controller. After confirmation of each ICMP packet receipt, the *F_RTT_* was calculated and averaged at the end of the session.

**Figure 6:**
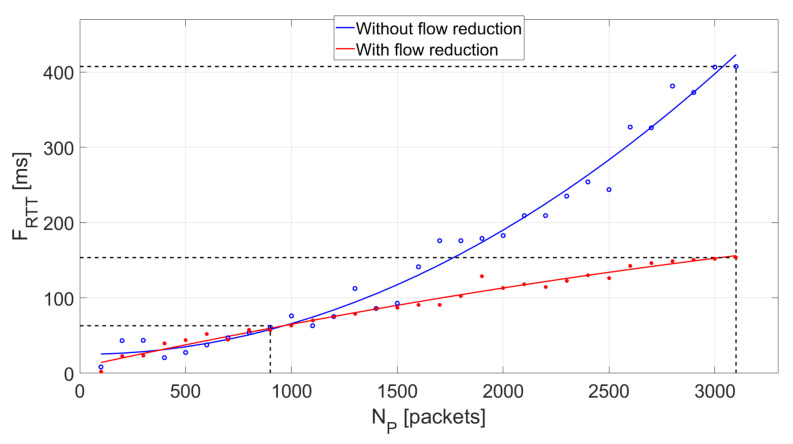
Effect of using flow granularity reduction for *I_F_* = 30,000 (packets/s) and *N_P_* < 3100.

The impact of a number of generated packets *N_P_* on the metric *F_RTT_* with the flow granularity reduction mechanism on and off is presented in Figure 6.

The flow granularity reduction mechanism does not affect the *F_RTT_* value if the number of generated packets is relatively small (*N_P_* < 900). However, if the mechanism is off, along with an increase in the number of packets loading the controller, *F_RTT_* increases rapidly. The mechanism contributes to a significant reduction in flow transfer delay, which also translates into a reduced controller load. 

**Figure 7:**
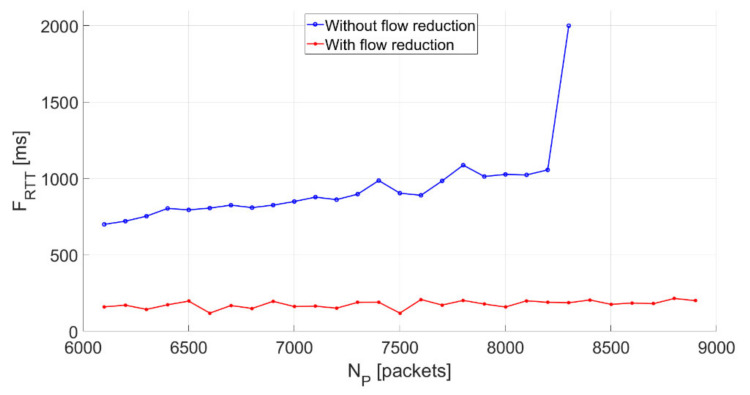
Effect of using flow granularity reduction for *I_F_* = 30,000 (packets/s) and *N_P_* > 6000.

Without the flow granularity reduction mechanism, a further increase in the number of packets transferred to the controller (Figure 7) leads to overloading, which blocks the introduction of new flow rules.

If the flow granularity reduction mechanism is on, traffic flooding is significantly limited. *F_RTT_* remains low ($F_{RTT}≅176.92\;$ ms) regardless of the number of incoming packets. This confirms the purposefulness of using the mechanism when flows are introduced in reactive mode. This mechanism protects the SDN controller from flooding traffic that might be a form of DoS attack. The in granularity was introduced only for a specific period, and this information was saved to the repository, which enabled us to constantly monitor the activity in the SDN.

### 4.4. MADMAS Evaluation

**Figure 8:**
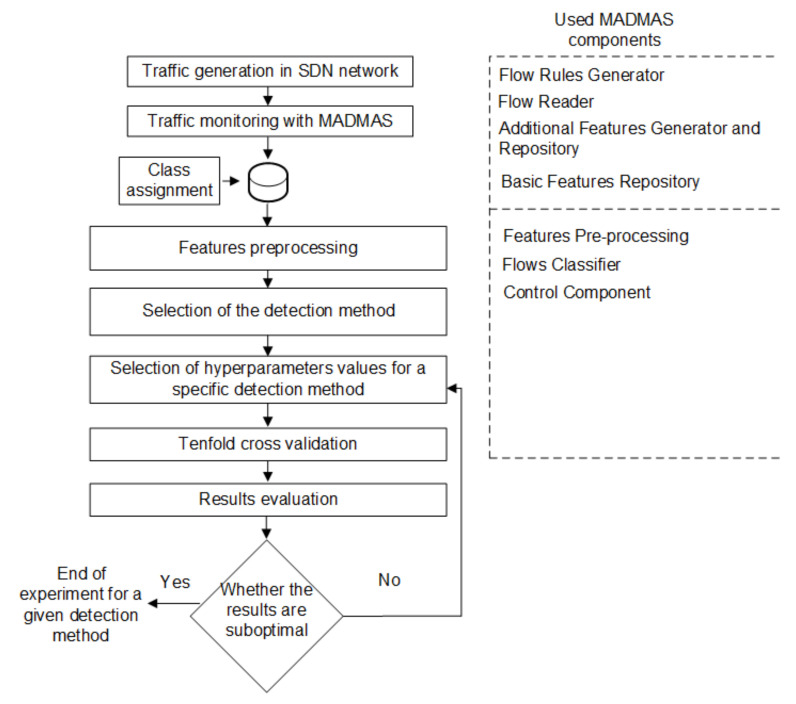
Test procedure.

The effectiveness of MADMAS in malicious flow detection was examined in the testbed environment (see Figure 5) following the procedure shown in Figure 8. 

The procedure started with the generation of both normal and malicious traffic. The user hosts generated requests to servers while malicious hosts launched attacks using the tools specified in Table 3. 

The MADMAS components acquired and processed the information sent to the controller from the data layer and created a set of input vectors X that were stored in the repository for further processing. Labels that defined the traffic class were assigned to the saved vectors for system validation. The traffic class was determined during its generation based on features that were not used in detection, e.g., a time stamp of traffic generation, IP address, etc. Then, pre-processing of features was performed, the classification technique under investigation was selected, and the values of its configuration parameters (see Table 2) were determined. Furthermore, tenfold cross-validation was performed, followed by evaluation of the obtained results. The procedure supports changing of the configuration parameters values to obtain the best flow classification results. The MADMAS components, used at specific stages of the procedure, are also shown in the right part of Figure 8.

The initial phase of the experiment focused on the selection of techniques with the best ability to detect SDN flows. The obtained results, presented in Table 4, confirmed the usefulness of using SVM, k-NN and HLVQ classifiers in the MADMAS system. For better clarity, fields with the best TPR, PPV, and AET values are marked in colour in Table 5. The advantage of these techniques over others is that all types of flows generated by hostile hosts are detected, especially DoS, P, and A_PG_ attacks.

However, the use of data mining in a real SDN environment requires its quick reaction to undesirable flows. The lowest values of the A_ET_ metric were achieved for LVQ1 and HLVQ1 classifiers, while the other classifiers had large A_ET_ values which, taking into account small TPR and PPV values, indicates their low usefulness in the considered application. By reducing the number of features by using principal component analysis (PCA) transformation [46] in the FPP, a significant reduction in A_ET_ was achieved for K-NN, HLVQ, and SVM classifiers, i.e., 2.0-, 1.8-, and 1.3-fold, respectively. The use of PCA transformation resulted in only a slight increase in the TPR and PPV values (Table 4), which confirms the low sensitivity of these techniques to reduce the number of features used. Therefore, we decided to use k-NN, HLVQ, and SVM techniques for further tests of the MADMAS system.

During the main phase of the experiment, the efficiency of the MADMAS system in the detection and classification of malicious flows was compared against selected alternative mechanisms (see Table 6) depicted in [29,31,36].

**Figure 9:**
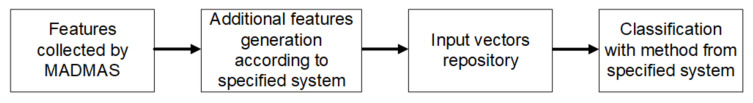
Implementation of alternative solutions.

For such a comparison to be credible, the alternative solutions should use the data collected by MADMAS from SDN flows, which are then processed and classified according to a specific concept, as shown in Figure 9. However, in our study, we used ready-made comprehensive solutions. Neither of these uses application layer features for classification purposes.

As shown in Table 7, in all the cases considered, the MADMAS system was better able to detect malicious flows compared to other solutions. The best metric values for each attack class are shown in colour for better visibility.

The better efficiency of the MADMAS system is particularly evident in the case of the access-by-exploit attack class, for which the following increments of classification performance metrics were obtained compared to other methods:

- For TPR by 31.4%;
- For PPV by 27.3%;
- For F1 by 29.3%.

This confirms the purposefulness of data acquisition from the application layer and the use of those data for flow classification.

The best results of malicious flow classification were obtained for the MADMAS system based on the SVM, especially in the case of DoS and APG attacks. The MADMAS system is less effective in detecting probe attacks but is still significantly better compared to other solutions. This is because of the similarity of probe attacks to normal traffic, which uses low-intensity port scanning and covert scanning.

To demonstrate the impact of using application layer features on the effectiveness of flow classification, an additional experiment was performed based on the solution proposed by [31]. System 2 was modified to enable the use of ICA-based application layer features stored in the MADMAS repository. The results obtained with and without application layer features are presented in Table 8.

The inclusion of the data obtained from the application layer in the solution proposed by Bhargava et al. [31] results in a slight improvement in the efficiency of the flow classification, with a simultaneous slight increase in execution time. We would like to emphasise that more in-depth experiments should be performed for the complete assessment of such impact. In particular, the relationship between the transformation of the ICA-based application layer features and the machine learning technique used should be identified.

**Figure 10:**
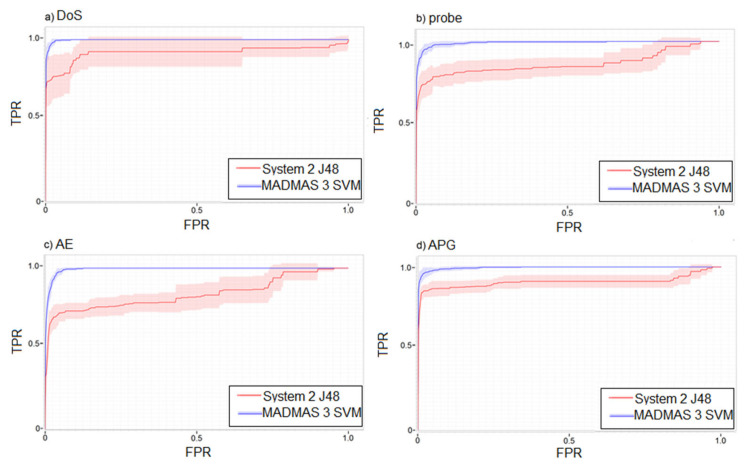
ROC curves.

The ROC curves, shown in Figure 10, confirm a much better classification performance of the MADMAS system than the solution described in [29] for all the considered threats. The biggest difference was for probe and access-by-exploit attacks (Figure 10b,c), while the best curve shape was obtained for DoS and APG attacks.

Although an SVM allows the MADMAS system to obtain high classification performance, its time efficiency, expressed by A_ET_, is much lower compared to the system based on HLVQ. This indicates the advisability of using an HLVQ-based system in SDNs with limited hardware resources (e.g., RAM, processor performance).

## 5. Conclusions

In this paper, we described the promising concept of using data mining techniques for the detection and classification of malicious flows in the SDN data plane, with a focus on the presentation of flow rule generation and flow classification mechanisms. The MADMAS system was implemented in a testbed environment, and its performance metrics were evaluated and compared with some alternative solutions.

The use of a virtual test environment with the SDN emulator and the existing SDN controller allowed testing the system in conditions similar to real ones. The experiments confirmed that the MADMAS system provides good flow detection performance for all types of malicious activities, in particular, probe and access-by-exploit attacks. The implementation flow granularity reduction prevents flooding traffic being passed to the SDN controller.

We also examined some classification techniques and assessed their applicability for malicious flow detection in the SDN data plane. The obtained results indicate that the use of the SVM for flow classification in the MADMAS system gives the best results in terms of classification performance. However, due to its low time efficiency, HLVQ seems to be a more appropriate solution for an SDN with limited hardware resources.

All MADMAS components are software-based; therefore, the system can easily be extended with additional procedures for flow generation and/or classification. The system architecture enables the identification and mitigation of threats caused by malicious actions in both fixed and wireless networks. However, a full assessment of the system effectiveness in detecting malicious flows requires further studies, especially in a real SDN. The OpenFlow-based SDN environment, depicted in [47] and which was developed with a specific focus on validation of SDN security mechanisms, could be successfully used for the MADMAS examination.

## Figures and Tables

**Table 1:** Comparison of selected concepts of malicious flow detection.

Concept	Diagnostic Features	Detection Technique	Detected Attacks
Revisiting traffic anomaly detection using software-defined networking [25]	Maximum entropy detector, TRW-CB, rate-limiting, NETAD	Predefined detection threshold	DoS L3–L4, probe
A fuzzy logic-based information security management for software-defined networks [26]	TRW-CB, rate-limiting	Fuzzy logic	DoS L3–L4
InMon corporation’s sFlow: a method of monitoring traffic in switched and routed networks [28]	TRW-CB, entropy level	Predefined detection threshold	DDoS, probe, malware propagation
Lightweight DDoS flooding attack detection using NOX/OpenFlow [29]	Flow’s parameters and its additional features	Self-organising map	DDoS L3–L4
Efficient anomaly detection and mitigation in a software-defined networking environment [30]	Flow’s parameters and its additional features	Decision tree J48	DoS, probe, U2R, U2L
Flexible network-based intrusion detection and prevention system on software-defined networks [34]	Parameters determined on the basis of captured packets	Decision tree C4.5	DoS, probe
Deep learning approach for network intrusion detection in software-defined networking [36]	Flow’s parameters	Deep learning neural network	DoS, probe, U2R, U2L
An approach for SDN traffic monitoring based on big data techniques [37]	Flow’s parameters	Not used; the approach focuses on real-time data collection	DoS
A DDoS attack mitigation scheme in ISP networks using machine learning based on SDNs [38]	Flow’s parameters	*K*-nearest neighbours	DDoS
InSDN: a novel SDN intrusion dataset [39]	Flow’s features	Not applied	DoS, DDoS, botnet, web attacks, brute force attacks, malware, probe, exploitation

**Table 2:** Modifiable parameters of malicious flow detection techniques.

Detection Technique	Parameter
MLP	$P_{hidden}$: number of hidden layers $P_{layers}$: number of neurons in layers m: momentum coefficient η: learning coefficient $P_{learning}$: number of learning cycles
RBF	$P_{C}$: number of clusters $P_{StdDev}$: minimum standard deviation of clusters
MSOM	$P_{high}$: height of the neuron ma_p_ $P_{width}$: width of the neuron map k: neighbourhood radius η: learning coefficient $P_{learning}$: number of learning cycles
LVQ, HLVQ	$P_{CV}$: number of codebook vectors η: learning coefficient $P_{learning}$: number of learning cycles
SVM	C: penalty factor γ: kernel function coefficient ξ: tolerance factor $P_{kernel}$: type of kernel function: radial sigmoid polynomial
k-NN	k: number of nearest neighbours $P_{distance}$: distance metric

**Table 3:** Traffic application tools.

Traffic Class	Applications
N	FTP, SSH, SMB, Apache, Web, Tomcat, RMI Ruby, Java RMI, Postgres, Telnet
DoS	Metasploit, Hping3, Nping
P	Metasploit, Nping
A_E_	Metasploit
A_PG_	Metasploit, Hydra

**Table 4:** Generated traffic parameters.

Traffic Class	Distribution Parameters	Subclass Count	Statistics of the Basic Features μ (Mean); σ (Deviation)		
			Packet Count	Byte Count	Flow Duration (s)
Normal	*k* = 1	20	*μ* = 74.16	*μ* = 128.046	*μ* = 6.50
	*λ* = 3		*σ* = 1025.32	*σ* = 2150.267	*σ* = 28.81
Probe	*μ* = 4000	7	*μ* = 0.07	*μ* = 6.06	*μ* = 2.30
	*σ* = 200		*σ* = 0.50	*σ* = 61.55	*σ* = 1.60
A_PG_	*μ* = 600	6	*μ* = 6.56	*μ* = 603.80	*μ* = 7.12
	*σ* = 200		*σ* = 5.09	*σ* = 702.72	*σ* = 3.82
DoS	*μ* = 1200	6	*μ* = 18.34	*μ* = 21.127	*μ* = 4.29
	*σ*= 200		*σ* = 89.85	*σ* = 171.170	*σ* = 3.21
A_E_	*μ* = 120	10	*μ* = 4.80	*μ* = 262.40	*μ* = 1.65
	*σ* = 40		*σ* = 15.538	*σ* = 651.04	*σ* = 0.50

**Table 5:** Efficiency of selected techniques in SDN flow detection.

Classification Technique	TPR				PPV				AET
	DoS	Probe	A_E_	A_PG_	DoS	Probe	A_E_	A_PG_	(s)
MSOM	0.48	0.96	0.05	0.78	0.61	0.95	0.66	0.83	45
LVQ1	0.80	0.96	0.08	0.83	0.68	0.96	0.37	0.89	6.3
MLP	0.99	0.97	0.04	0.85	0.83	0.96	0.04	0.91	302
RBF	0.99	0.97	0.08	0.83	0.99	0.96	0.08	0.82	218
k-NN	0.99	0.99	0.69	0.98	0.99	0.99	0.76	0.96	74
HLVQ1	0.99	0.98	0.48	0.95	0.98	0.98	0.67	0.95	3.1
SVM	0.99	0.99	0.65	0.98	0.99	0.99	0.83	0.97	46,6
k-NN with PCA	0.99	0.99	0.68	0.98	0.99	0.97	0.77	0.96	36.3
HLVQ1with PCA	0.96	0.98	0.50	0.95	0.92	0.98	0.72	0.96	1.7
SVM with PCA	0.99	0.99	0.62	0.99	0.99	0.99	0.83	0.97	35.8

**Table 6:** Compared systems.

System	Classification Technique	Feature Vector Configuration
System 1 [29]	SOM neural network	Appropriate for each concept: Basic features; Additional features.
System 2 [31]	J48 decision tree	
System 3 [36]	Deep learning ANN	
MADMAS_1	k-NN	Basic features; Additional features; Application layer feature transformation based on independent component analysis.
MADMAS _2	HLVQ	
MAMDAS_3	SVM	

**Table 7:** Summary of the results.

System	Metric	DoS	Probe	A_E_	A_PG_
System 1	TPR	0.34	0.46	0.26	0.78
	PPV	0.52	0.64	0.51	0.77
	F1	0.41	0.54	0.35	0.78
	A_ET_	980 ms			
System 2	TPR	0.84	0.75	0.59	0.88
	PPV	0.82	0.78	0.72	0.86
	F1	0.83	0.77	0.65	0.87
	A_ET_	8030 ms			
System 3	TPR	0.73	0.67	0.50	0.89
	PPV	0.81	0.80	0.65	0.90
	F1	0.59	0.64	0.49	0.85
	A_ET_	9100 ms			
MADMAS_1	TPR	0.91	0.92	0.86	0.97
	PPV	0.97	0.83	0.97	0.99
	F1	0.94	0.87	0.91	0.98
	A_ET_	2690 ms			
MADMAS_2	TPR	0.87	0.78	0.76	0.96
	PPV	0.95	0.80	0.88	0.97
	F1	0.91	0.79	0.82	0.96
	A_ET_	440 ms			
MADMAS_3	TPR	0.97	0.78	0.86	0.98
	PPV	0.99	0.96	0.99	0.99
	F1	0.98	0.86	0.92	0.99
	A_ET_	5550 ms			

**Table 8:** Impact of the ICA-based application layer features on the effectiveness of System 2.

System	Metric	DoS	Probe	A_E_	A_PG_
System 2 without ICA-based application layer features	TPR	0.84	0.75	0.59	0.88
	PPV	0.82	0.78	0.72	0.86
	F1	0.83	0.77	0.65	0.87
	A_ET_	8030 ms			
System 2 with ICA-based application layer features	TPR	0.86	0.79	0.63	0.89
	PPV	0.83	0.78	0.76	0.87
	F1	0.84	0.77	0.68	0.88
	A_ET_	9080 ms			
